# Draft genome sequence of *Dethiobacter alkaliphilus* strain AHT1^T^, a gram-positive sulfidogenic polyextremophile

**DOI:** 10.1186/s40793-017-0268-9

**Published:** 2017-09-21

**Authors:** Emily Denise Melton, Dimitry Y. Sorokin, Lex Overmars, Alla L. Lapidus, Manoj Pillay, Natalia Ivanova, Tijana Glavina del Rio, Nikos C. Kyrpides, Tanja Woyke, Gerard Muyzer

**Affiliations:** 1 Department of Freshwater and Marine Ecology, Microbial Systems Ecology, Institute for Biodiversity and Ecosystem Dynamics, University of Amsterdam, Amsterdam, The Netherlands; 2 Winogradsky Institute of Microbiology, Research Centre of Biotechnology, RAS, Moscow, Russia; 3 Department of Biotechnology, Delft University of Technology, Delft, The Netherlands; 4 Center for Algorithmic Biotechnology, Institute of Translational Biomedicine, St. Petersburg State, University, St. Petersburg, Russia; 5 Joint Genome Institute, Walnut Creek, CA USA; 6 Biological Data Management and Technology Center, Lawrence Berkeley National Laboratory, Berkeley, CA USA; 7 Department of Biological Sciences, Faculty of Science, King Abdulaziz University, Jeddah, Saudi Arabia

**Keywords:** Extreme environment, Soda lake, Sediment, Haloalkaliphilic, Gram-positive, *Firmicutes*

## Abstract

*Dethiobacter alkaliphilus* strain AHT1^T^ is an anaerobic, sulfidogenic, moderately salt-tolerant alkaliphilic chemolithotroph isolated from hypersaline soda lake sediments in northeastern Mongolia. It is a Gram-positive bacterium with low GC content, within the phylum *Firmicutes*. Here we report its draft genome sequence, which consists of 34 contigs with a total sequence length of 3.12 Mbp. *D. alkaliphilus* strain AHT1^T^ was sequenced by the Joint Genome Institute (JGI) as part of the Community Science Program due to its relevance to bioremediation and biotechnological applications.

## Introduction

Soda lakes are formed in environments where high rates of evaporation lead to the accumulation of soluble carbonate salts due to the lack of dissolved divalent cations. Consequently, soda lakes are defined by their high salinity and stable highly alkaline pH conditions, making them dually extreme environments. Soda lakes occur throughout the American, European, African, Asian and Australian continents and host a wide variety of *Archaea* and *Bacteria*, specialized at surviving under such high salt and high pH conditions [1]. These haloalkaliphiles drive a number of biogeochemical cycles essential to their survival, most notably; the sulfur cycle is very active in these unique habitats [2–4]. The most noteworthy taxa associated with the reductive sulfur cycle are the 10.1601/nm.3456 and the 10.1601/nm.3874. Recently, a number of Gram-positive 10.1601/nm.3874 genomes have been analyzed and published describing their metabolic potential and environmental adaptations, including the polyextremophile 10.1601/nm.11479 [5], and species belonging to the 10.1601/nm.4329 spp. [6–8] and the 10.1601/nm.4325 spp. [9]. Here we give an extended insight into the first known genome of a haloalkaliphilic Gram-positive sulfur disproportionator within the phylum 10.1601/nm.3874: 10.1601/nm.13712 AHT1^T^.

## Organism information

### Classification and features

**Fig. 1:**
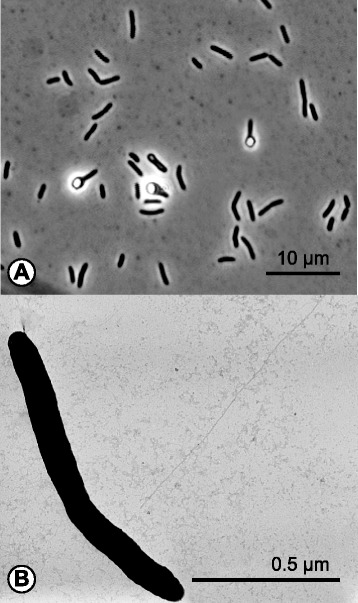
Morphology of *D. alkaliphilus* AHT1^T^. **a** Phase contrast micrograph of cells. **b** Electron microscope image of a *D. alkaliphilus* AHT1^T^ cell

**Fig. 2:**
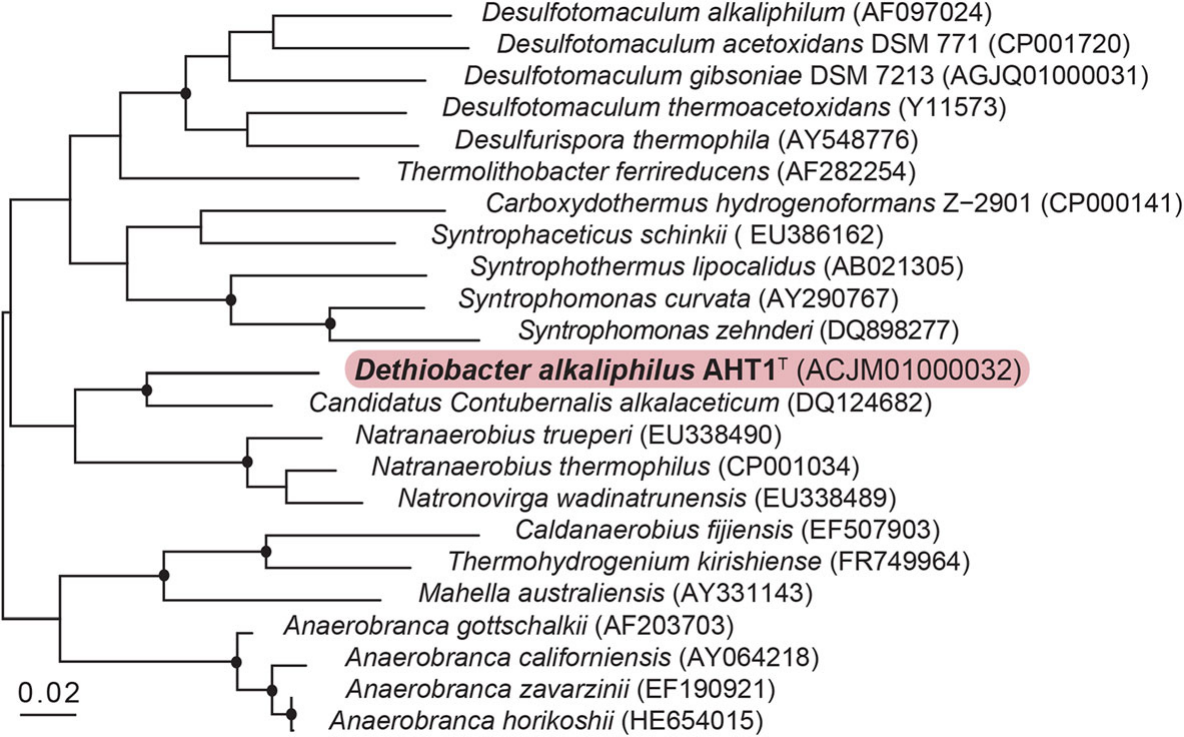
Neighbour-joining tree based on 16S rRNA gene sequences showing the phylogenetic position of *D. alkaliphilus* AHT1^T^ to other species within the phylum *Firmicutes*. The *Deltaproteobacteria* were used as an outgroup, but were pruned from the tree. The dots indicate bootstrap values between 80 and 100%. The scale bar indicates a 2% sequence difference. The tree was constructed with the ARB software package [48] and the SILVA database [29]. The bootstrap values were calculated using MEGA-6 [49]

The haloalkaliphilic anaerobe 10.1601/nm.13712 AHT1^T^ was isolated from hypersaline soda lake sediments in northeastern Mongolia [10]. 10.1601/nm.13712 AHT1^T^ cells are Gram-positive and the motile rod-shaped cells form terminal ellipsoid endospores (Fig. 1). The strain tolerates salt concentrations ranging from 0.2–0.8 M Na^+^ with an optimum at 0.4 M and is an obligate alkaliphile, growing within a pH range from 8.5–10.3 with an optimum at 9.5 [10]. Phylogenetic analysis showed that strain AHT1^T^ is a member of the phylum 10.1601/nm.3874 and the order 10.1601/nm.3876 (Fig. 2). Its closest relative is an acetate-oxidizing syntrophic alkaliphile, described as “*Candidatus* Contubernalis alkalaceticum” which was isolated from a soda lake [11] (Fig. 2). The 16S ribosomal RNA of 10.1601/nm.13712 AHT1^T^ (EF422412) is 88% identical to the 16S rRNA of “*Candidatus* Contubernalis alkalaceticum” (DQ124682) [12].

### Extended feature descriptions

10.1601/nm.13712 AHT1^T^ is an obligate anaerobe that can produce sulfide by using elemental sulfur and polysulfides as electron acceptor [10]. Additionally, it has been shown to incompletely reduce thiosulfate to sulfide and sulfite with hydrogen or formate as electron donor [10]. Strain AHT1^T^ is the first representative from the 10.1601/nm.3874 with the metabolic capacity to grow by elemental sulfur disproportionation [13] and, therefore, is a very interesting organism to compare to the typical sulfur disproportionators from the 10.1601/nm.3456. This species may play an important role in the reductive sulfur cycle in soda lake environments [2] and possibly also in other alkaline anaerobic habitats, such as serpentinization “cement springs”, where sequences closely related to 10.1601/nm.13082 have been found [14, 15]. Also, its affiliation with the syntrophic 10.1601/nm.3875 “*Candidatus* Contubernalis alkalaceticum” (Fig. 2) implies that 10.1601/nm.13712 AHT1^T^ could be involved in syntrophic anaerobic metabolic activity. More classifications and features of this species are listed in Table 1.

**Table 1:** Classification and general features of 10.1601/nm.13712 AHT1^T^

MIGS ID	Property	Term	Evidence code
	Classification	Domain: *Bacteria* Phylum: 10.1601/nm.3874 Class: 10.1601/nm.3875 Order: 10.1601/nm.3876 Family: 10.1601/nm.4462 Genus: 10.1601/nm.13082 Species: 10.1601/nm.13712 Type strain: AHT1^T^	TAS [51] TAS [52–54] TAS [55, 56] TAS [57, 58] TAS [59] TAS [10, 60] TAS [10, 60] TAS [10]
	Gram stain	positive	TAS [10]
	Cell shape	rod-shaped	TAS [10]
	Motility	motile	TAS [10]
	Sporulation	endospore-forming	TAS [10]
	Temperature range	mesophile	TAS [10]
	Optimum temperature	33	
	pH range; Optimum	8.5-10.3; 9.5	TAS [10]
	Carbon source	CO_2_, acetate	TAS [10]
MIGS-6	Habitat	hypersaline soda lakes, sediments	
MIGS-6.3	Salinity	moderately salt-tolerant	
MIGS-22	Oxygen requirement	anaerobe	
MIGS-15	Biotic relationship	free-living	
MIGS-14	Pathogenicity	none	
MIGS-4	Geographic location	northeastern Mongolia; lakes Hotontyn and Shar-Burdiin	TAS [2]
MIGS-5	Sample collection	September 1999	
MIGS-4.1	Latitude	48° 19′ 40″	TAS [2]
MIGS-4.2	Longitude	114° 30′ 16″	TAS [2]
MIGS-4.4	Altitude	1000 m	

Evidence codes - *IDA* Inferred from Direct Assay, *TAS* Traceable Author Statement (i.e., a direct report exists in the literature); *NAS* Non-traceable Author Statement (i.e., not directly observed for the living, isolated sample, but based on a generally accepted property for the species, or anecdotal evidence). These evidence codes are from the Gene Ontology project [Cite ontology project]

## Genome sequencing information

### Genome project history

This organism was selected for sequencing at the JGI (http://jgi.doe.gov) based on its potential for bioremediation and biotechnological applications. It is part of the Community Science Program: Haloalkaliphilic sulfate-, thiosulfate- and sulfur-reducing bacteria (CSP_788492). The project is registered in the Genomes OnLine Database (Ga0028528) [16] and the permanent draft genome sequence is deposited in GenBank (RefSeq: NZ_ACJM00000000.1). Draft sequencing and assembly were performed at the JGI using state of the art sequencing technology [17]. The project information is summarized in Table 2.

**Table 2:** Project information

MIGS ID	Property	Term
MIGS 31	Finishing quality	permanent draft
MIGS 28	Libraries used	Solexa
MIGS 29	Sequencing platforms	454
MIGS 31.2	Fold coverage	33.2
MIGS 30	Assemblers	Newbler, (2.0.00.20-PostRelease-11-05-2008-gcc-3.4.6), PGA [23], VELVET [22]
MIGS 32	Gene calling method	Prodigal [28]
	Locus Tag	DealDRAFT
	Genbank ID	ACJM00000000
	Genbank Date of Release	12.12.2013
	GOLD ID	Gp0001962
	BIOPROJECT	PRJNA30985
	Project relevance	bioremediation, environmental biotechnology

### Growth conditions and genomic DNA preparation

Strain AHT1^T^ was grown anaerobically at 30 °C in Na-carbonate buffered mineral medium (22 g/L Na_2_CO_3_, 8 g/L NaHCO_3_, 6 g/L NaCl, 1 g/L K_2_HPO_4_) with a pH of 10 and 0.6 M total Na^+^. Additionally, 4 mM NH_4_Cl, 1 mM MgCl_2_ x 6H_2_O and 1 mlL^−1^ trace element solution were added [18]. After sterilization, acetate serving as carbon source (2 mM) and thiosulfate (20 mM) the electron-acceptor, were also added to the medium. The culture (2 L) was grown in a 10 L bottle mounted on a magnetic stirrer whereby the headspace (8 L) was replaced by 100% (*v*/v) H_2_, at 0.5 Bar overpressure, acting as the electron-donor. Half the culture volume (1 L) was centrifuged at 13,000 g for 30 min, the pellet was washed with 1 M NaCl and frozen at -80 °C until further downstream processing. DNA was extracted from the pellet by the phenol-chloroform method after pre-treatment with SDS-proteinase K according to Marmur [19]. The concentration and molecular weight of the DNA were checked by UV spectroscopy and gel electrophoresis, respectively.

### Genome sequencing and assembly

The size of the assembled 10.1601/nm.13712 AHT1^T^ genome sequence was 3.12 Mbp. The draft genome was generated at the JGI using a combination of Sanger, Solexa/Illumina [20] and 454 DNA sequencing technologies [21]. An 8 Kb Sanger library was constructed that provided 2.5 x coverage of the genome (15,321 reads generated) and a Solexa shotgun library and a 454 Titanium standard library, which provided 25× genome coverage totalling 110.0 Mbp of 454 data. The 454 Titanium data were assembled with Newbler. The Newbler consensus sequences were computationally shredded into 2 Kb overlapping fake reads (shreds). Illumina sequencing data was assembled with VELVET, version 1.0.13 [22], and the consensus sequences were computationally shredded into 1.5 Kb overlapping fake reads (shreds). We then integrated Sanger reads, the 454 Newbler consensus shreds and the Illumina VELVET consensus shreds using the PGA assembler [23], to combine sequence data from all three platforms for a most contiguous assembly. The software Consed [24] was used in the computational finishing process as described previously [25]. The final draft assembly contained 34 contigs in 5 scaffolds.

### Genome annotation

The assembled sequence was automatically annotated with the JGI prokaryotic annotation pipeline [26] with additional manual review using the IMG-ER platform [27]. Genes were predicted using Prodigal [28], ribosomal RNAs were detected using models built from SILVA [29] and tRNAs were predicted with tRNAScanSE [30]. The predicted CDs were translated and used to search the NCBI non-redundant database UniProt, TIGRFam, Pfam, KEGG, COG and InterPro databases. The final annotated genome is available from the IMG system [31]. We performed a CheckM analysis [32] and assessed that the genome is 95.8% complete.

## Genome properties

The genome is 3,116,746 bp long with a GC content of 48.46%. A total of 3213 genes were found, of which 3163 coded for proteins and 50 genes encoded only RNA. From the total genes, 69.19% was assigned a putative function. The IMG taxon ID is 643,886,183. The different functional gene groups are summarized in Table 3. Furthermore, the number of genes assigned to functional COG categories is displayed in Table 4.

**Table 3:** Nucleotide content and gene count levels of the genome

Attribute	Value	% of total
Genome size (bp)	3,116,746	100
DNA coding (bp)	2,773,015	88.97
DNA G + C (bp)	1,510,353	48.46
DNA scaffolds	34	100
Total genes	3213	100
Protein coding genes	3163	98.44
RNA genes	50	1.56
Pseudo genes	0	0
Genes in internal clusters	177	not reported
Genes with function prediction	2223	69.19
Genes assigned to COGs	1971	61.34
Genes with Pfam domains	2632	81.92
Genes with signal peptides	170	5.29
Genes with trans-membrane helices	962	29.94
CRISPR repeats	0	0

**Table 4:** Number of genes associated with general COG functional categories

Code	Value	% of total	Description
J	175	7.89	Translation, ribosomal structure and biogenesis
A	not reported	not reported	RNA processing and modification
K	134	6.04	Transcription
L	83	3.74	Replication, recombination and repair
B	1	0.05	Chromatin structure and dynamics
D	45	2.03	Cell cycle control, cell division, chromosome partitioning
V	58	2.62	Defense mechanisms
T	131	5.91	Signal transduction mechanisms
M	124	5.59	Cell wall/membrane biogenesis
N	52	2.35	Cell motility
U	34	1.53	Intracellular trafficking and secretion
O	90	4.06	Posttranslational modification, protein turnover, chaperones
C	178	8.03	Energy production and conversion
G	81	3.65	Carbohydrate transport and metabolism
E	227	10.24	Amino acid transport and metabolism
F	69	3.11	Nucleotide transport and metabolism
H	149	6.72	Coenzyme transport and metabolism
I	80	3.61	Lipid transport and metabolism
P	133	6.00	Inorganic ion transport and metabolism
Q	24	1.08	Secondary metabolites biosynthesis, transport and catabolism
R	183	8.25	General function prediction only
S	129	5.82	Function unknown
–	1242	38.66	Not in COGs

The total is based on the number of protein coding genes in the genome

## Insights from the genome sequence

### Extended insights: Metabolic potential

**Fig. 3:**
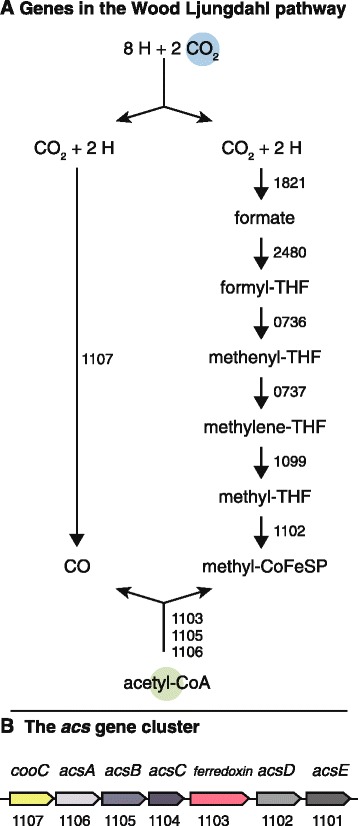
**a** KEGG orthologs annotated in the gene pathway encoding Wood Ljungdahl inorganic carbon fixation in *D. alkaliphilus* strain AHT1^T^. **b** The *acs* gene cluster with locus tags. All locus tag numbers are indicated and preceded by DealDRAFT_

**Fig. 4:**
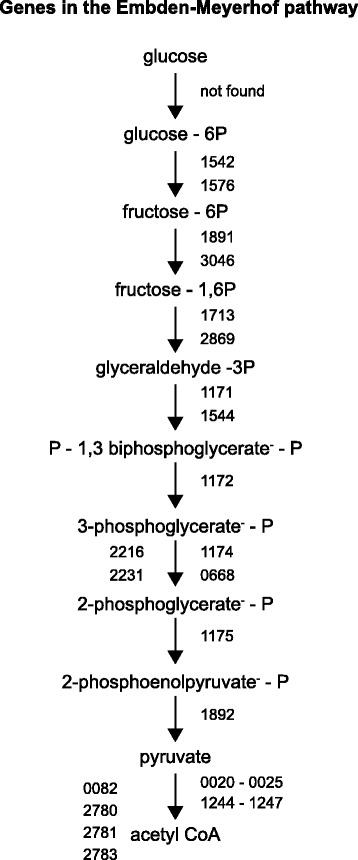
KEGG orthologs annotated in the Embden-Meyerhof pathway of organic carbon assimilation in *D. alkaliphilus* strain AHT1^T^. The numbers of the locus tags of the genes catalyzing each reaction are indicated and must be preceded by DealDRAFT_

Hydrogen metabolism requires a number of hydrogenase operons, including the *hyd* operon, and a Ni-Fe metallocenter assembly (*hyp*) [33]. The first part of the hydrogenase *hyd* operon is the small hydrogenase subunit *hydA* located at DealDRAFT_1217, the closest NCBI BLAST hit [12] of this protein is the *hydA* gene in 10.1601/nm.4338 (Desgi_1397) with 70.4% similarity in a pair-wise alignment [34]. Directly adjacent to *hydA*, is the large subunit *hydB* (DealDRAFT_1218) in the 10.1601/nm.13712 AHT1^T^ genome. This subunit is most similar (75.9%) to the *hydB* subunit in 10.1601/nm.4315 sp. UNSWDHB (UNSWDHB_1527) [12, 34]. DealDRAFT_1219 is a cytochrome B561 of 198 amino acids and could therefore be the interacting partner and gamma subunit *hydC* in the *hyd* operon. The 6-gene *hyp* operon *hypABCDEF* is responsible for the assemblage of the Ni-Fe uptake hydrogenases [35]. The last 5 proteins of the *hyp* operon are annotated in the 10.1601/nm.13712 AHT1^T^ genome (DealDRAFT_0838-DealDRAFT_0842) and follow the organization *hyp*BFCDE, as has been seen before in 10.1601/nm.1279 [36]. The first gene in the operon (DealDRAFT_0843) is a hypothetical protein of 88 nucleotides length and is assigned to pfam01155 *hypA*, which is 42.6% identical to the *hypA* gene in 10.1601/nm.4533 *thermoaceticum*. Therefore, this hypothetical protein is most likely *hypA* in 10.1601/nm.13712 AHT1^T^. Using hydrogen as electron donor, 10.1601/nm.13712 AHT1^T^ can grow autotrophically by fixing inorganic carbon through the Wood Ljungdahl pathway, the key genes are all present in the genome (Fig. 3a), including the *acs* gene cluster (Fig. 3b). Heterotrophic growth by 10.1601/nm.13712 AHT1^T^ can be achieved with glucose and fructose [10], the entire glycolysis pathway is present in the genome (Fig. 4). Carbohydrate metabolism in 10.1601/nm.13712 AHT1^T^ also includes oxidation of short chain organic acids; the tetrameric pyruvate oxidoreductase is present in the conformation *porBADC* (DealDRAFT_1244 – DealDRAFT_1247). Lactate dehydrogenases could not be found, although there is an L-lactate permease (DealDRAFT_0239), an L-lactate transport protein (DealDRAFT_1845) and a large and small subunit acetolactate synthase (DealDRAFT_2169 and *2170*). For assimilation of acetate, strain AHT1^T^ has an acetyl coenzyme A synthetase (DealDRAFT_1887).

10.1601/nm.13712 AHT1^T^ might play a role in the reductive sulfur cycle in alkaline habitats since it grows as a thiosulfate and sulfur/polysulfide reducer or by sulfur disproportionation in laboratory cultures [10]. The genome sequence contains a thiosulfate sulfurtransferase (DealDRAFT_1917), which is located directly adjacent to another sulfur transferase (Rhodanese domain DealDRAFT_1918). Both alpha and beta subunits of the adenylylsulfate reductase *apr* operon were also found (DealDRAFT_1379, DealDRAFT_1380). The *qmo* electron transfer complex, which usually accompanies the *apr* operon [37], is not found. Key sulfur reduction genes such as *sat* (sulfate reduction), *dsr* (sulfite reduction) and *psr* (sulfur reduction) were also not found in this draft genome. As 10.1601/nm.13712 AHT1^T^ can reduce and disproportionate elemental sulfur/polysulfide in laboratory cultures [10, 13], the absence of these genes is surprising. It is conceivable however, that the sequencing quality of the permanent draft is insufficient to recover complete pathways. Indeed, CheckM analysis revealed that the genome was only 95.8% complete. Unfortunately, we can therefore not explain the key dissimilatory disproportionation mechanism from this genomic data. The genome also contains some assimilatory sulfate reduction genes, such as *cysND* (DealDRAFT_1193 and DealDRAFT_1192).

### Extended insights: Haloalkaliphilic adaptations

**Fig. 5:**
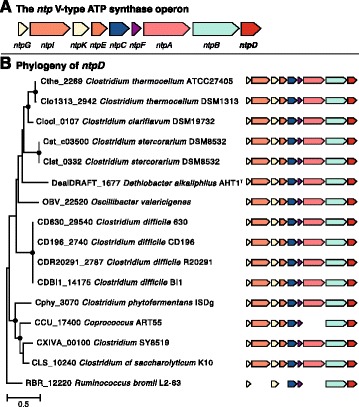
**a** The *ntp* Vacuole-type ATP synthase operon structure. **b** 93 *ntpD* homologs (DealDRAFT_1677) within the genus *Clostridia* were aligned in Clustal Omega [34] and an unrooted neighbour-joining tree was generated in MEGA-6 [49]. From this tree, we picked the branch that contained the *D. alkaliphilus* AHT1^T^ *ntpD* sequence and computed a new neighbourjoining tree with gene DCR20291_1119 as an outgroup. The scale bar indicates a 0.5% sequence difference and conserved gene neighbourhoods of those genes were investigated using MGcV [50]. Large dots at the tree nodes indicate a bootstrap value of >85 (1000 replicates)

**Fig. 6:**
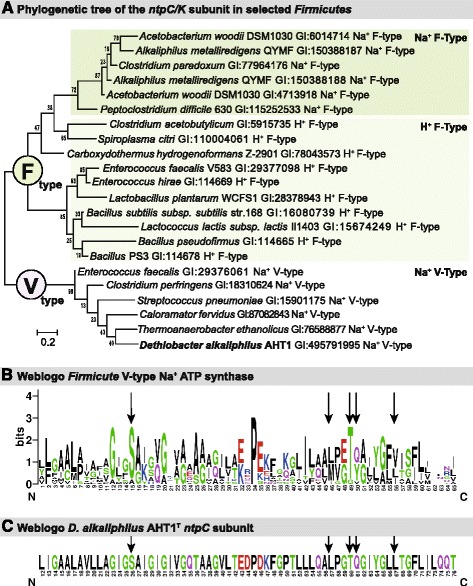
**a** Phylogeny of the F- vs. V-type ATPase within the *Firmicutes*. Numbers on the tree nodes indicate bootstrap values (1000 replicates). Scale bar indicates 0.2% sequence difference. **b** Weblogo of conserved region within the *ntpC/K Firmicu* subunit [42, 43]. **c** Weblogo of aligned *D. alkaliphilus* AHT1^T^ subunit *ntpC* (DealDRAFT_1683) where conserved Na^+^ binding regions (in B and C) are indicated with black arrows

In order to generate ATP, 10.1601/nm.13712 AHT1^T^ has an *ntp* gene operon encoding a vacuolar ATP synthase (V_0_V_1_-type) (DealDRAFT_1677 – DealDRAFT_1685) (Fig. 5a). This operon structure is conserved among the 10.1601/nm.3875 (Fig. 5b). The *ntp* operon encodes the ATP synthase for ATP generation and follows the GILEXFABD organization in the 10.1601/nm.503 phylum [38]. In the 10.1601/nm.3874, the gene organization is slightly different at GIKECFABD (Fig. 5a, b). In 10.1601/nm.13712 AHT1^T^ these genes are located from DealDRAFT_1685 (*ntpG*) to DealDRAFT_1677 (*ntpD*). The *ntpD* subunit within the operon is annotated as being of the V-type. In order to confirm that the ATP synthase is indeed V-type [39], we constructed a phylogenetic tree of the transmembrane *c/K* subunits of 10.1601/nm.3874 known specifically to be V- or F-type [40] and NCBI annotation] and aligned the 10.1601/nm.13712 AHT1^T^ *ntpC* sequence (DealDRAFT_1683) with these other sequences (Fig. 6a) [41]. As seen before, there was a clear separation between V-type and F-type ATP synthase, where the AHT1^T^ sequence clustered together with the V-type ATP synthase. In addition, the sequences are tentatively clustered into separate H^+^ or Na^+^ coupled ATPase branches. The AHT1^T^ sequence was positioned within a Na^+^ coupled V-type ATP synthase group, indicating that this organism’s ATP synthase is coupled specifically to Na^+^ translocation across the membrane. In order to explore this further, we looked at specific Na^+^ binding residues and ligands on the transmembrane *c/K* subunit [40], and created a Weblogo for the Na^+^ specific 10.1601/nm.3874 V-type ATP synthase (Fig. 6b) [42, 43]. When we aligned the *ntpC* sequence of 10.1601/nm.13712 AHT1^T^ we found that it contains all the conserved five amino acids (Ser26, Leu57, Thr60, Gln61 and Tyr64) specific for Na^+^ translocation [40] (Fig. 6c). Thus, the 10.1601/nm.13712 AHT1^T^ genome contains a Na^+^ coupled V-type ATP synthase.

In order to import protons to retain the intracellular pH, the genome contains the multi-subunit electrogenic sodium/proton antiporter *mrp* (DealDRAFT_2487–2497), that pumps protons into the cell and sodium out of the cell [44]. To retain osmotic balance, 10.1601/nm.13712 AHT1^T^ has numerous substrate binding regions and transporters for glycine betaine (e.g. DealDRAFT_2378, _2380 and DealDRAFT2842, _2844), leading to the conclusion that osmoprotectants are used to maintain cellular turgor pressure, instead of the salt-in strategy. Another necessity for alkaliphilic bacteria is to prevent proton leakage from cells, which they can achieve through structural membrane adaptations [1]. The genome contains the genes to synthesize the squalene precursors dimethylallyl diphosphate and isopentenylallyl diphosphate through the non-mevalonate pathway [45]. The accompanying locus tags within the KEGG non-mavalonate pathway (M00096) are *dxs* (DealDRAFT_0731), *dxr/ispC* (DealDRAFT_2409), *ispD* (DealDRAFT_2331), *ispE* (DealDRAFT_2584), *ispF* (DealDRAFT_2332), *ispG* (DealDRAFT_2411) and *ispH* (DealDRAFT_0659). However, we did not find genes similar to *hpnCDE*, which function in the formation of squalene from its precursors [46]. Thus, 10.1601/nm.13712 AHT1^T^ does not seem to have this membrane adaptation to haloalkaline environments, although it could also be due to the incompleteness of the genome. Nevertheless, it has been shown that 10.1601/nm.4934 C-125, also a *Firmicute*, survives in the haloalkaline environment by increased levels of acidic polymers in its cellular membrane resulting in a cell wall negative charge [47]. It is possible that 10.1601/nm.13712 AHT1^T^ supports a similar mechanism to survive the alkaline pH values of its environment.

## Conclusions

In this manuscript we globally characterize the genome of 10.1601/nm.13712 AHT1^T^, which was isolated from hypersaline soda lakes sediment in north-eastern Mongolia. Investigation of the genome of this anaerobic sulfidogen identified genes for the Wood Ljungdahl pathway (autotrophic growth, Fig. 3) and the Embden-Meyerhof pathway (heterotrophic growth Fig. 4). Thus the carbon metabolism of this microbe is fairly versatile. 10.1601/nm.13712 AHT1^T^ is capable of disproportionation in laboratory cultures, thus future genomic analyses with qPCR may provide insights into the disproportionation of sulfur compounds. 10.1601/nm.13712 AHT1^T^ is well adapted to the haloalkaline environment, we found genes for active energy generation with a sodium V-type ATP synthase (Fig. 6). In addition, transporters for the osmoprotectants glycine and betaine were found to maintain cellular homeostasis and protection from the saline external environment. Further research will extend our knowledge on the ecophysiology of haloalkaliphiles, their role in nutrient cycling in extreme environments and their adaptations to this polyextreme environment. Moreover, insight in the genome sequence and subsequent transcriptomic or proteomic analysis will be helpful to infer the potential role of 10.1601/nm.13712 AHT1^T^ in the biotechnological removal of sulfur compounds from wastewater and gas streams.

## Abbreviations

F-type: Phosphorylation factor-type
IMG: Integrated Microbial Genomes
IMG-ER: Integrated Microbial Genomes - Expert Review
JGI: Joint Genome Institute
NCBI: National Center for Biotechnology Information
THF: tetrahydrofolate
V-type: Vacuole-type
